# First Data on the (Poly)phenolic Profiling of Farmacista Honorati Persimmon Fruit (*Diospyros kaki* Thunb.) at Commercial Harvest and after Treatments for Astringency Removal

**DOI:** 10.3390/plants13131768

**Published:** 2024-06-26

**Authors:** Lapo Renai, Daniele Bonetti, Giulia Bonaccorso, Francesca Tozzi, Stefania Nin, Edgardo Giordani, Massimo Del Bubba

**Affiliations:** 1 Department of Chemistry “Ugo Schiff”, University of Florence, Via della Lastruccia 3, 50019 Sesto Fiorentino, Italy; 2 Council for Agricultural Research and Economics, Research Centre for Vegetable and Ornamental Crops, 51017 Pescia, Italy; 3 Department of Agriculture, Food, Environment and Forestry (DAGRI), University of Florence, 50019 Sesto Fiorentino, Italy

**Keywords:** (poly)phenols, functional food, soluble tannins, targeted metabolomics, liquid chromatography, tandem mass spectrometry

## Abstract

This study aims to provide the first report on the soluble and polyphenolic profiles of “Farmacista Honorati” (FH) persimmons, which is a marketed cultivar with no existing data on its nutraceutical value. Total soluble tannins (TSTs) and major soluble (poly)phenols in FH fruits before and after post-harvest commercial treatments with carbon dioxide and ethylene were analyzed. Fruits at commercial harvest had a TST content of 1022 ± 286 mg GAL/100 g d.w. Whereas, after deastringency treatments, an 85% and 83% reduction were observed for carbon dioxide- and ethylene-treated fruits, respectively. Carbon dioxide treatment resulted in the insolubilization of tannins around comparable values in most fruit cultivars, despite the variable soluble tannin content in untreated fruit. By targeted metabolomic profiling, nineteen (poly)phenolic substances were quantified in the investigated untreated and treated fruits. Gallic acid (99 mg/100 g d.w.), (+)-catechin (1.8 mg/100 g d.w.), ellagic acid (1.2 mg/100 g d.w.), and (−)-epicatechin (1.1 mg/100 g d.w.) were the predominant compounds in the untreated FH samples. After the application of post-harvest treatments, a non-nutraceutical relevant decrease of 8-19% in the targeted (poly)phenolic content was generally observed. Ethylene induced the most significant reduction in the individual (poly)phenolic compounds in the FH fruits.

## 1. Introduction

Persimmon (*Diospyros kaki* Thunb.) is a commercially relevant fruit crop whose world production amounted to approximately 4.2 million tons in 2020 [1], mainly in the Asian regions, which cover about 95% of the total production. However, the production of persimmon fruits is also important in Europe, where its cultivation is essentially confined to Spain (around 490,000 tons) and Italy (around 48,000 tons), mainly with the genotypes “Kaki Tipo” and, above all, “Rojo Brillante” [2].

Persimmon fruits are generally acknowledged as an exceptional source of primary (e.g., sugars and ascorbic acid) and secondary metabolites [3]. Among the latter group, (poly)phenolic compounds (e.g., benzoic acids, flavanols, and flavonols) are considered a very important nutraceutical trait of kaki, potentially involved in health-protection mechanisms against various diseases [3,4,5].

(Poly)phenolic compounds of persimmon fruits, and particularly a wide group of gallic acid derivatives like tannins, are also very important in influencing fruit palatability [4]. In fact, persimmon fruits may exhibit a different astringent character depending on their genetics, which regulates the characteristics and amount of tannins present in the fruit pulp. Additionally, their genetic trait is responsible for the production by the seeds of volatile compounds capable of insolubilizing the tannins, thus decreasing the fruit astringency [3,6]. In this regard, persimmon varieties are divided into Pollination Constant Astringent (PCA), Pollination Variant Astringent (PVA), Pollination Variant Non-Astringent (PVNA), and Pollination Constant Non-Astringent (PCNA) types, and only the fruits of PCNA and pollinated PVNA cultivars are not astringent at harvest time [3,7]. Conversely, PCA, PVA, and parthenocarpic PVNA fruits are edible only after deastringency processes, commonly obtained through their exposure to carbon dioxide or ethylene [3,8], which gives rise to a strong decrease in soluble tannins and an increase in palatability [9,10].

In recent years, there have been efforts to increase the knowledge of the (poly)phenolic composition of persimmon fruits. A targeted comparative analysis of individual native phenolic compounds in the fresh fruit pulp of various kaki cultivars revealed the presence of common traits among astringent and non-astringent cultivars from Italy, Japan, and China [11,12,13,14]. Specifically, flavanols such as catechin, epicatechin, and epigallocatechin, as well as hydroxybenzoic acids including vanillic acid and ferulic acid, were identified as prevalent metabolites. Conversely, gallic acid has been reported as a predominant phenolic compound in astringent varieties [11,12,13,14,15], whilst it was not detected in some non-astringent genotypes [13] or found at concentrations lower than other phenolics [14]. Other phenolic compounds, such as certain hydroxycinnamic acids (e.g., chlorogenic and caffeic acids), quercetin, and phloridzin, have been sporadically studied and identified in various persimmon cultivars from China and Italy [12,13]. Several other phenolic acids, flavanols, and flavonols, as well as some of their derivatives, were putatively identified in the Rojo Brillante cultivar through an untargeted analytical approach using liquid chromatography (LC) coupled via electrospray interface (ESI) with time-of-flight (TOF) or ion-trap mass spectrometry (MS) [16,17,18]. Recently, the occurrence of high concentrations of ellagic acid has also been reported in persimmon pulp from Rojo Brillante and Kaki Typo fruits grown in Italy, further highlighting its nutraceutical potential [6]. The genotype effect on the (poly)phenolic profile and content in Rojo Brillante and Kaki Typo varieties was also recently demonstrated, evidencing the crucial role of the cultivar in determining the occurrence of these secondary metabolites in persimmon fruits [6]. However, in most cases, a limited set of persimmon varieties has been investigated compared to the numerous genotypes described in literature through the analysis of a few (poly)phenolic compounds [19].

Among the persimmon genotypes never investigated for their (poly)phenolic composition pattern, the “Farmacista Honorati” (FH) variety stands out. This genotype has been cultivated in the Misilmeri area (Palermo, Italy, geographical coordinates 38°01′12.7″ N–13°27′04.0″ E) since the early 18th century in specialized orchards grafted onto *Diospyros virginiana* plants [20] and is now under valorization by the Sicily Region. The FH cultivar belongs to the PCA group and therefore its fruits are astringent at harvest, thus requiring a post-harvest treatment with ethylene [21] or carbon dioxide [22] before consumption.

Based on the aforementioned considerations, this paper aims to provide the first time data on the soluble (poly)phenolic fraction of FH persimmons pulp at commercial harvest and after post-harvest treatments with carbon dioxide and ethylene. In detail, FH fruits untreated and treated for deastringency removal will be characterized by (i) their soluble tannin content and (ii) a set of 37 major (poly)phenolic metabolites, thus outlining (the absent) information on the secondary metabolic profile and nutraceutical relevance of this unique cultivar and contributing to its marketing success.

## 2. Results and Discussion

### 2.1. TST Content of FH Fruits

**Figure 1:**
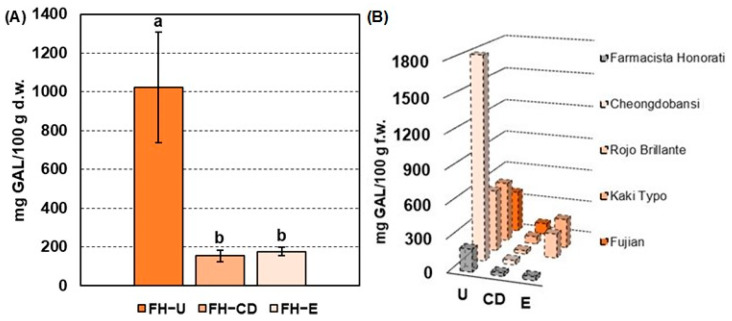
(**A**) Mean concentration values (*n* = 3, standard deviation in brackets) of total soluble tannins (TSTs) expressed in mg GAL/100 g d.w. in Farmacista Honorati (FH) fruits at commercial harvest (FH-U) and after carbon dioxide (FH-CD) and ethylene (FH-E) deastringency treatments. Different letters mark a statistical difference (*p*-value < 0.05) according to the Wilcoxon signed-rank test. (**B**) TST content of FH fruits (mg GAL/100 g f.w.) compared to data in the literature of different persimmon cultivars before and after deastringency treatments. References - Cheongdobansi: Chung, H. S. et al. 2015. Rojo Brillante & Kaki Typo: Del Bubba M. et al. 2009. Fujian: Liu J. et al. 2018.

The mean values of the total soluble tannin (TST) assay performed on FH-U (i.e., at commercial harvest), FH-CD (i.e., after carbon dioxide treatment), and FH-E (i.e., after ethylene treatment) are reported in Figure 1A. In detail, FH-U fruits were characterized by a TST content of 1022 ± 286 mg GAL/100 g d.w., which is in line with other persimmon astringent cultivars (e.g., Mopan and Deabong) exhibiting a soluble tannin content of about 1200 mg GAL/100 g [23,24,25,26]. Nevertheless, FH-U fruits are characterized by a halved TST content when compared with European and Rojo Brillante and Kaki Typo varieties containing up to 2700–2750 mg GAL/100 g d.w. [10]. 

After deastringency treatments, FH fruits were characterized by a significant reduction in the TST content (Figure 1A), up to 154 ± 29 mg GAL/100 g d.w. (about 85% of reduction) and 177 ± 22 mg GAL/100 g d.w. (about 83% of reduction) after carbon dioxide (CD) and ethylene treatments (E), respectively. Figure 1B shows the trend of TST reduction (mg GAL/100 g f.w.) after CD and/or E deastringency treatments found in previous research on different astringent persimmon cultivars. It is interesting to note that astringency removal using carbon dioxide produces insolubilization of tannins at comparable values in most fruit cultivars despite the variable content in untreated fruits. In detail, Cheongdobansi and Rojo Brillante fruits were characterized by a TST content of about 40 mg GAL/100 g f.w. and 31 mg GAL/100 g f.w., respectively, being close to the values obtained for FH-CD fruits (30.8 mg GAL/100 g f.w., Figure 1B). A milder tannin insolubilization was observed for the Kaki Typo (~67 mg GAL/100 g f.w.) and Fujian (~100 mg GAL/100 g f.w.) varieties but was always in line with the deastringency trend observed for FH fruits. On the other hand, the ethylene-based treatments seem to produce a more homogeneous percentage of tannin insolubilization, depending on the initial TST values. Rojo Brillante fruits, indeed, were characterized by a TST reduction of 86%, similar to the trend observed for FH-E fruits (i.e., 83%), whilst Kaky Typo persimmons were less affected by this deastringency treatment [10,27,28]. As a general outcome, commercially treated FH fruits have a soluble polyphenolic fraction similar to other cultivars. Finally, the TST content of untreated and treated FH fruits points out the bioactivity potential of these extracts, being compatible with the TST values of extracts of other varieties of Diospyros kaki (e.g., Fuyu and Rojo Brillante) characterized by high protein quenching, antioxidant, and neuroprotective properties [5,29,30].

### 2.2. Targeted (Poly)phenol Profiling in FH Fruits

Simple and condensed/polymerized (poly)phenols (mainly including hydroxybenzoic acids, flavanols, and proanthocyanidins) are co-present in persimmons [3]. However, even if proanthocyanidin polymers (i.e., tannins) can promote antioxidant and radical scavenging activities [31], their degree of polymerization influences and/or limits gastric absorption by human epithelium cells [32]. Thus, to evaluate the nutraceutical potential of the “available” tannin fraction, simple (poly)phenols were studied in FH fruits before and after de-stringency treatments.

#### 2.2.1. (Poly)phenolic Compounds in FH Fruits at Commercial Harvest

Table 1 shows the mean concentration values of the target (poly)phenolic compounds investigated by LC-MS/MS analysis in the FH methanolic extracts.

FH-U fruits exhibited much higher concentrations of “available” (poly)phenolic compounds in comparison with the Rojo Brillante cultivar grown in similar environmental conditions (i.e., approximately 104 mg/100 g d.w. vs. about 44–54 mg/100 g d.w.) [11], thus highlighting the overall higher nutraceutical value of the FH variety.

Among the 37 investigated compounds, NCHL, CG, and PB2 were never detected in FH-U flesh. In detail, p-HYD, VAN, CRY, DCQ, SIN, PA2, PC1, QUE-GAL, QUE-RUT, QUE-RHA, KAM-GLU, KAM-RUT, PHL, LUT, and ESC were below their detection limits, whereas PRO and CAF ranged between the MDL and MQL. Thus, in FH-U fruit pulp, 17 analytes were found at concentrations higher than the MQLs (Table 1). Among them, GAL exhibited the highest concentration value, at about 99 mg/100 g d.w. The relevant content of GAL was already identified by other authors as a typical metabolic trait of astringent persimmons [11,13,33]. Interestingly, in FH-U fruits, the concentration of GAL was three and two times higher compared to astringent Kaki Typo and Rojo Brillante grown in Northern Italy, respectively [11]. Additionally, GAL in FH-U fruits was by far more concentrated also in comparison with the astringent Mopan variety (19 mg/100 g d.w) [12]. Among the other hydroxybenzoic acids, only SAL was quantified in FH-U persimmons, showing a concentration value of 33 ± 3 µg/100 g d.w.

Among the hydroxycinnamic acids, CHL was found to be the most abundant (90 ± 28 μg/100 g d.w.), followed by p-COU (20 ± 8 μg/100 g d.w.) and FER (5 ± 3 μg/100 g d.w.). The occurrence of these acids, except for p-COU, was confirmed also in astringent Chinese [13] and Italian varieties [11].

The target flavanols in FH-U persimmons exhibited an occurrence trend in the order CAT > EPI > >EGCG > GCG > EGC > ECG > PB1, with very different concentrations depending on the compound considered. In detail, in FH-U fruits, EPI and CAT were by far the predominant polyphenols of this group with 1107–1751 μg/100 g d.w., whereas the rest of the flavanols ranged between 10 μg/100 g d.w. (PB1) and 101 μg/100 g d.w. (EGCG). Interestingly, the only proanthocyanidin herein detected and quantified in FH-U was PB1, which is in accordance with the results obtained for astringent Kaki Typo and Rojo Brillante fruits [11]. Upon examination of the literature, CAT was found to be the predominant flavanol in the astringent persimmon genotype Mopan [5,12] as well as in the non-astringent Japanese persimmons varieties Maekawa-Jiro and Matsumoto-Wase-Fuyu [9], while in the cultivars Gapjubaekmok (astringent) and Hiratanenashi, Tone-Wase and Ishibashi-Wase (non-astringent), the main flavanol was EGC [14,33]. Overall, these data suggest that the astringent quality of persimmon does not appear to be closely associated with the relative abundance of these flavanols. The flavonols family was the least abundant among those included in this research, and only two (i.e., QUE and QUE-GLU) of the seven analytes studied here for this group of (poly)phenols were quantified in FH fruits. In detail, QUE and QUE-GLU were found at concentrations of 20 ± 1 μg/100 g d.w. and 14 ± 4 μg/100 g d.w., respectively. Analogous findings were reported for flavanols in Rojo Brillante fruits grown in Italy [11] and Spain [34] as well as untargeted identification protocols by time-of-flight mass spectrometry [16].

Two chalcones (i.e., PHL and PHL-GLU) were included among the targeted analytes. PHL was found below the MDL in the investigated cultivar, whereas the concentration value in FH-U persimmons of PHL-GLU was 42 ± 4 μg/100 g d.w. The results observed were significantly lower than the values reported elsewhere for astringent and non-astringent cultivars [11,13]. 

A further miscellaneous set of compounds was included in the target group of analytes based on the findings of previous studies [11,25]. This group comprised two coumarin derivatives (i.e., SCO and ESC), one flavone (i.e., LUT), and EA. Among them, SCO was quantified in FH-U (44 ± 11 μg/100 g d.w.), whereas ESC and LUT were not detected in both fruit varieties. EA was among the most abundant phenolic compounds in FH-U, with concentrations of about 1.2 mg/100 g d.w. This result is in accordance with the high EA concentrations found previously in Rojo Brillante and Kaki Typo fruits (about 6 mg/100 g d.w.) [11]. The occurrence of EA in persimmon pulp is a relatively new finding in foodomics, since the first report about its presence in D. kaki varieties dates back to 2019 [6], and this polyphenol is commonly considered a typical trait of other well-known functional foods, like raspberries, strawberries, and pomegranates [35,36,37]. The presence of EA in persimmons can be rationalized considering that its synthesis is related to gallic acid metabolism [38], which is very abundant in this fruit species, as mentioned above.

#### 2.2.2. Influence of Post-Harvest Treatments on (Poly)phenolic Profile

**Figure 2:**
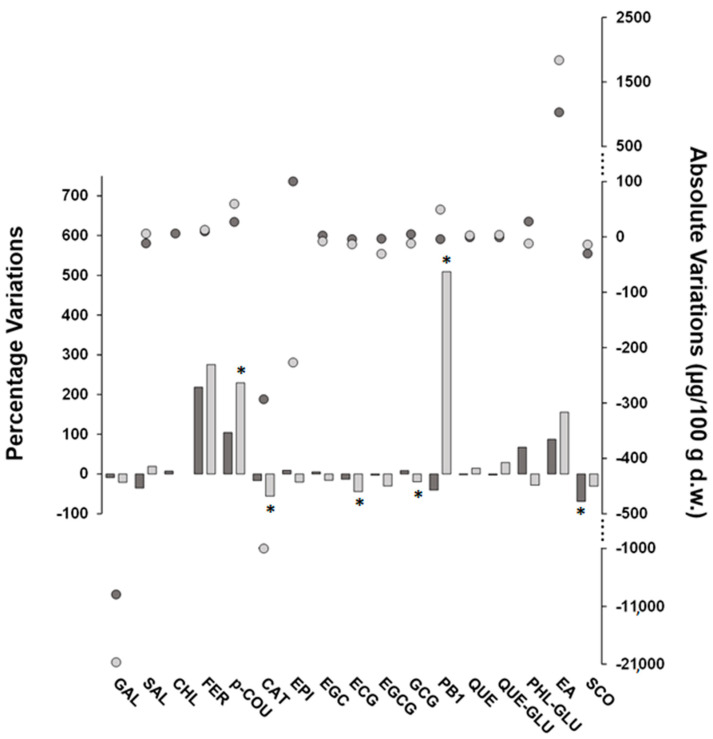
Percentage (bars) and absolute (round symbols) variations of concentration values (μg/100 g d.w.) of target (poly)phenolic compounds found in FH persimmons exposed to carbon dioxide (dark grey color) or ethylene (light grey color) as post-harvest treatments compared to fruits at commercial harvest (i.e., untreated). The bars marked with an asterisk refer to compounds with statistically different variations according to the Games–Howell multiple comparisons test (*n* = 3, α = 0.05). Note that for CHL, the variation due to ethylene treatment was not estimated due to the unquantifiable concentrations found in the fruits.

Post-harvest treatments induced a slight reduction in the (poly)phenol concentrations of FH fruits. In detail, the sum of the targeted (poly)phenols decreased in FH fruits exposed to carbon dioxide and ethylene by approximately 8% and 19%, respectively, resulting in a lower impact on the (poly)phenolic content of FH fruits compared to the results reported in the literature for other astringent varieties. In detail, reduction percentages of 30–60% and 30–85% of the total content of (poly)phenols were found by treating Kaki Typo, Rojo Brillante, and Mopan fruits with ethylene and carbon dioxide, respectively [11,39,40]. Although these post-harvest treatments give rise to metabolic effects in fruits involving multiple signaling-related genes that are difficult to rationalize, it should be noted that in our study, the concentrations of post-harvest treatment gases and/or the exposure time were lower than those reported elsewhere, thus suggesting a possible explanation of the findings observed here. Figure 2 illustrates the percentage and absolute variations for FH (poly)phenolic compounds in response to treatments with carbon dioxide and ethylene. Absolute changes have been added in order to ease the evaluation of the percentage variations data, thus more effectively contextualizing their meaning. Both post-harvest treatments resulted in variations in analyte concentrations, which, however, were statistically significant only in some cases. Interestingly, these variations appear to be compound-related rather than treatment-dependent. In fact, most of the compounds underwent the same increasing or decreasing trend with both treatments, with the main exception being PB1, which was significantly increased by ethylene and decreased by carbon dioxide, albeit not significantly. In accordance with data reported elsewhere [10], the compound-dependent changes of individual (poly)phenols during post-harvest treatments may be the result of complex transformation processes, which comprise metabolite interconversion leading to progressive insolubilization of (poly)phenols formerly present in soluble form as well as the degradation of the phenolic function. More specifically, the strong increase in PB1 after treatment with ethylene could be framed in the process of the conversion of monomeric flavanols into proanthocyanidins [27] and is in agreement with the observed decrease in CAT and EPI, which was indeed much higher than the PB1 increase on a molar basis. Indeed, the treatment with ethylene was the one that mostly influenced the native (poly)phenolic composition of the FH fruits, and, in many cases, the percentage variations due to the treatment with ethylene were higher than those found for the treatment with carbon dioxide and for some compounds were even statistically significant (i.e., p-COU, CAT, ECG, and GCG). Moreover, PRO, VAN, and CAF increased after ethylene treatment and became quantifiable while remaining below the MDL or MQL after exposure to carbon dioxide. Indeed, this latter treatment did not produce statistically significant concentration changes, with the exception of SCO, which exhibited a marked decrease (about 70%).

## 3. Materials and Methods

### 3.1. Chemicals

Ultra-pure water was obtained from a Milli-Q system (Millipore, Billerica, MA, USA). Acetone (HPLC grade) and Folin-Ciocalteu (F-C) reagent were purchased from Sigma-Aldrich (St. Louis, MO, USA). Sodium fluoride was obtained from Merck (Darmstadt, Germany). The following reference standards of (poly)phenolic compounds for ultra-high performance liquid chromatographic-tandem mass spectrometric (UHPLC-MS/MS) analysis were purchased from Sigma-Aldrich: gallic acid (GAL), protocatechuic acid (PRO), neochlorogenic acid (NCHL), 4-hydroxybenzoic acid (p-HYD), procyanidin B1 (PB1), epigallocatechin (EGC), (+)-catechin (CAT), chlorogenic acid (CHL), esculetin (ESC), caffeic acid (CAF), procyanidin B2 (PB2), vanillic acid (VAN), cryptochlorogenic acid (CRY), (−)-epigallocatechin gallate (EGCG), procyanidin C1 (PC1), (−)-epicatechin (EPI), (−)-gallocatechin gallate (GCG), p-coumaric acid (p-COU), (−)-epicatechin gallate (ECG), procyanidin A2 (PA2), (−)-catechingallate (CG), ferulic acid (FER), sinapic acid (SIN), salicylic acid (SAL), quercetin-3-galactoside (QUE-GAL), ellagic acid (EA), quercetin-3-rutinoside (QUE-RUT), quercetin-3-glucoside (QUE-GLU), phloretin-2′-glucoside (PHL-GLU), quercetin-3-rhamnoside (QUE-RHA), kaempferol-3-rutinoside (KAM-RUT), kaempferol-3-glucoside (KAM-GLU), quercetin (QUE), luteolin (LUT), and phloretin (PHL). The reference standards of scopoletin (SCO) and 1,5-dicaffeoylquinic (DCQ) acid were obtained from Extrasynthese (Genay, France). Mass spectrometry grade water, methanol, and formic acid (FoA) were obtained from Carlo Erba Reagents (Milan, Italy). The nylon membranes with a porosity of 0.2 µm, used for filtering the fruit extracts, were obtained from VWRTM International (Radnor, PA, USA). 

### 3.2. Persimmon Fruits Origin

Naturally parthenocarpic FH persimmons, cultivated in an orchard located in Misilmeri (Palermo, Italy, 38°01′12.7″ N and 13°27′04.0″ E) under rural practices compliant with the Integrated Pest Management Standards of the Sicily Region [18], were kindly supplied by the “Francesco Rizzo” SME (Misilmeri, Palermo, Italy). After commercial harvest (November 2020), one lot consisting of twelve fruits was stored at 4 °C, transported to the laboratory without undergoing any type of treatment (untreated samples, U), and immediately analyzed. 

### 3.3. Post-Harvest Treatments

Ethylene and carbon dioxide post-harvest treatments were performed by the “Francesco Rizzo” SME according to its own commercial procedures. Ethylene-based accelerated ripening was performed in gas-tight polypropylene containers and exposed to an atmosphere containing 2% CO_2_ and 2‰ SIOETIL (a gas mixture of 95% N2 and 5% ethylene) (Air Liquide Italia, Milan, Italy) for 48 h at a relative humidity (R.H.) of 70% and a temperature of 20 °C. Deastringency treatment was carried out by exposing the fruits to a 90% CO_2_ atmosphere for 24 h at a temperature ranging from about 15 to 20 °C. After the post-harvest treatments, a dozen FH fruits treated with carbon dioxide and ethylene were stored at 4 °C and then transported for immediate analysis. 

### 3.4. Samples and Extraction

Six FH fruits free of defects were selected from the groups of fruits untreated and treated with carbon dioxide and ethylene to prepare biological replicates (three replicates, each derived from two fruits). After peel and seed removal, the flesh of the fruits belonging to the same treatment was homogenized and frozen using liquid nitrogen and subsequently freeze-dried at −80 °C to a constant weight. The mean moisture content of all the samples is reported in Table S1 of the Supplementary Materials. Before targeted LC-MS/MS analysis, the (poly)phenolic fraction was investigated by analyzing the total soluble tannins (TSTs) occurring in fruits before and after deastringency treatments. TST analysis was performed using the F-C method (see Section 3.5) on the fruit extracts obtained, as elsewhere reported [5,19] with slight modifications. In detail, 180 mg of freeze-dried FH (-U, -CD, and -E) fruit were mechanically mixed for 10 min with 15 mL of a methanol/water solution 8/2 (*v*/*v*), containing 10 mM NaF to inactivate polyphenol oxidase. The resulting heterogeneous mixture was centrifuged at 1800× *g* for 5 min and the supernatant was collected. This procedure was repeated three times to evaluate the total phenolics recovered from the fruit matrix. By TST analysis, it was possible to evaluate that two consecutive extractions allowed for the recovery of more than 90% of the total content of (poly)phenols in all the investigated conditions. Hence, for the TSP and LC-MS/MS analyses, the first and second extractions were combined and concentrated using a rotavapor in order to remove the organic solvent. Before LC-MS/MS analysis, the resulting aqueous extracts, obtained under the optimal conditions, were filtered onto 0.2 µm nylon membranes and stored in the dark at 4 °C. 

### 3.5. TST Analysis

The TSTs were analyzed spectrophotometrically using the method proposed by Gao et al. [15], slightly modified using GAL as the reference standard. A proper volume (5–10 µL) of the extracts, to obtain an absorbance value included in the calibration curve, was mixed with 500 µL of F-C reagent. After 5 min of incubation in the dark at room temperature (23–25 °C), 2.5 mL of a 1 M sodium carbonate solution was added; the mixture obtained was brought to 5 mL volume with ultra-pure water. Once the solution was incubated for 1 h, the absorbance was measured at 760 nm. The results are expressed in mg of GAL per 100 g on a dry weight basis (mg GAL/100 g d.w.).

### 3.6. UHPLC-MS/MS Analysis

The LC system (Shimadzu, Kyoto, Japan) included a low-pressure gradient quaternary pump (Nexera X2 LC-30AD), a thermostatted column compartment (CTO/20AC), an autosampler (SIL-30AC), a degassing unit (DGU-20A 5R), and a module controller (CBM-20A). A 5500 QTrap™ mass spectrometer (Sciex, Toronto, ON, Canada) with a Turbo V™ interface carrying an electrospray (ESI) probe was hyphenated to the LC system. Chromatographic analysis was conducted based on a method previously optimized [25], using an Acquity BEH C18 column (15 cm × 2.1 cm i.d.; particle size 1.7 μm) (Waters, Milford, MA, USA) equipped with an Acquity UHPLC BEH C18 Van Guard column (5 mm × 2.1 mm, 1.7 μm; Waters, Milford, MA, USA). The eluents for UHPLC-MS/MS analysis were the following: eluent A—mixture 99.95/0.05 (*v*/*v*) of MS grade water and FoA; eluent B—mixture 99.95/0.05 (*v*/*v*) of LC-MS grade methanol and FoA. To carry out chromatographic analysis, the column temperature was set at 55 °C and the following gradient was used: 0–1 min isocratic 4% B, 1–25.5 min linear gradient 4–90% B, 25.5–27.0 min isocratic 90% B. The flow rate was 0.3 mL/min, and the injection volume was 5 μL. The LC-MS/MS analysis was conducted using the Multiple Reaction Monitoring (MRM) method with ESI in negative ionization mode. The most intense transition was used for quantification (quantifier) and the second most intense transition was used to confirm the identification (qualifier) of each analyzed compound. The compound-dependent parameters were fine-tuned by directly infusing properly diluted target analyte standard solutions. The source-dependent parameters were optimized using flow injection analysis at the optimal LC flow and mobile phase composition. A detailed description of the source and compound-dependent parameters (Table S2) as well as the preparation of the standard stock solutions and the figures of merit of the analytical method (Table S3) are reported in the Supplementary Materials.

NCHL, PB2 and CG, which were found to be absent in the investigated samples, were chosen as surrogate standards due to the low availability of isotopically labelled polyphenol reference standards. The recovery (R%) of the method was evaluated by spiking a representative sample with 150 µL of 1 mg/L solution of NCHL, PB2, and CG. The matrix effect percentage (ME%) was investigated by spiking the extract aliquots with a diluted solution of surrogate standards in the linearity range and then comparing the slope of the surrogate standard curves in the matrix and the solvent. For each surrogate standard, R% values were calculated by correcting the standard areas for ME and then by comparing the concentrations obtained in the extracts and the solvent. Table 2 shows the values of R%, ME%, and the method detection and quantitation limits for the surrogate standards.

### 3.7. Data Analysis

Data plots were created using Microsoft Office Excel 365 (Microsoft Corporation, Redmond, WA, USA). The Wilcoxon signed-rank test of the R package stats was used to evaluate the differences among the TST values of the untreated and treated FH fruits. Multiple comparison tests of the mean concentration values of the target (poly)phenols were performed using the Games–Howell nonparametric test (Minitab^®^ 17.1.0, Minitab Inc., State College, PA, USA), allowing for a comparison of the mean values and standard deviations without assuming equal variances. 

## 4. Conclusions

This study offers unique information on the (poly)phenolic composition of FH persimmons, thus providing the first nutraceutical assessment of this cultivar. TST analysis evidenced that the post-harvest treatments for astringency removal for FH fruits produce a soluble (poly)phenolic fraction that is comparable in quality to other marketed cultivars, placing FH persimmons at the same nutraceutical level for consumers. By LC-MS targeted profiling, 17 (poly)phenolic compounds representative of the major categories of secondary metabolites occurring in FH fruits were quantified. Due to the high content of some (poly)phenolic metabolites (e.g., GAL, EA, CAT, and EPI), FH persimmons can be addressed as functional foods with a relevant nutraceutical value, again comparable or higher than other cultivars marketed on a large scale. Moreover, the changes in the (poly)phenolic profile of FH fruits after carbon dioxide and ethylene post-harvest treatment for commercial purposes were assessed for the first time, highlighting moderate variations. In particular, the CO_2_ treatment evaluated here turned out to be more conservative, since it kept most of the investigated phenols available in soluble form. Ethylene treatment, even though it influenced the soluble (poly)phenolic metabolites the most, strongly promoted the occurrence of some of them (e.g., VAN, CAF, and PB1).

## Figures and Tables

**Table 1:** Mean values (*n* = 3, standard deviation in brackets) of the concentrations (μg/100 g d.w.) of major soluble (poly)phenols in FH fruits at commercial harvest (FH-U) and after deastringency treatments using carbon dioxide (FH-CD) and ethylene (FH-E).

	FH-U	FH-CD	FH-E
**Hydroxybenzoic acids**			
**GAL**	99,238 (23,633)	90,339 (27,784)	78,597 (21,221)
**PRO**	<3 ^b^	<1 ^a^	17 (3)
**p-HYD**	<1 ^a^	<1 ^a^	<1 ^a^
**VAN**	<11 ^a^	<11 ^a^	435 (65)
**SAL**	33 (3)	21 (11)	39 (10)
**Total**	**99,271**	**90,360**	**79,088**
**Hydroxycinnamic acids**			
**CHL**	90 (28)	97 (48)	<47 ^b^
**CRY**	<2 ^a^	<2 ^a^	<2 ^a^
**CAF**	<13 ^b^	<3 ^a^	22 (8)
**DCQ**	<0.5 ^a^	<0.5 ^a^	<0.5 ^a^
**FER**	5 (3)	15 (6)	18 (9)
**p-COU**	26 (9)	53 (24)	86 (4)
**SIN**	<4 ^a^	<4 ^a^	<4 ^a^
**Total**	**121**	**165**	**126**
**Flavanols**			
**CAT**	1751 (120)	1458 (302)	781 (148)
**EPI**	1107 (271)	1208 (484)	881 (264)
**EGC**	50 (5)	53 (5)	42 (1)
**ECG**	30 (2)	26 (3)	16.5 (0.3)
**EGCG**	101 (20)	98 (19)	7 (12)
**GCG**	60 (4)	65 (1)	49 (4)
**PB1**	10 (6)	6 (3)	59 (12)
**PA2**	<3 ^a^	<3 ^a^	<3 ^a^
**PC1**	<8 ^a^	<8 ^a^	<8 ^a^
**Total**	**3109**	**2914**	**1836**
**Flavonols**			
**QUE**	20 (2)	20 (1)	23 (7)
**QUE-GLU**	14 (4)	14 (6)	18 (8)
**QUE-GAL**	<1 ^a^	<1 ^a^	<1 ^a^
**QUE-RUT**	<1 ^a^	<1 ^a^	<1 ^a^
**QUE-RHA**	<3 ^a^	<3 ^a^	<3 ^a^
**KAM-GLU**	<1 ^a^	<1 ^a^	<1 ^a^
**KAM-RUT**	<1 ^a^	<1 ^a^	<1 ^a^
**Total**	**34**	**34**	**41**
**Chalcones**			
**PHL**	<1 ^a^	<1 ^a^	<1 ^a^
**PHL-GLU**	42 (4)	70 (26)	30 (6)
**Total**	**42**	**70**	**30**
**Others**			
**EA**	1183 (277)	2213 (933)	3022 (1058)
**LUT**	<2 ^a^	<2 ^a^	<2 ^a^
**ESC**	<5 ^a^	<5 ^a^	<5 ^a^
**SCO**	44 (11)	14 (5)	30 (9)
**Total**	**1227**	**2227**	**3052**

^a^ method detection limit; ^b^ method quantitation limit.

**Table 2:** Recovery (R%, mean and SD in brackets, *n* = 3) and matrix effect percentage (ME%) for NCHL, PB2, and CG in FH samples. Method detection (MDL, µg/100 g d.w.) and quantitation (MQL, µg/100 g d.w.) limits are reported for each surrogate standard.

Surrogate Standards	R% *	ME%	MDL	MQL
NHCL	115 (20)	−18	3.1	14.0
PB2	71 (8)	−40	0.2	0.9
CG	124 (19)	−9	0.5	1.9

* Spiked concentration 25 μg/L.

## Data Availability

The original contributions presented in the study are included in the article/Supplementary Material. Further inquiries can be directed to the corresponding author/s.

## Supplementary Materials

The following supporting information can be downloaded at https://www.mdpi.com/article/10.3390/plants13131768/s1, Table S1: Moisture content of Farmacista Honorati (FH) samples, freeze-dried for 72 h.; Table S2: Optimized compound-dependent parameters for the investigated compounds; Figure S1: MRM reconstructed LC-MS/MS chromatogram of a standard mixture (100 µg/L) of the investigated (poly)phenols; Table S3: Values of instrumental ILOD and ILOQ (μg/L), %RSD values of peak area (250 μg/L), and linearity data.
