# Supplementary material for: First Data on the (Poly)phenolic Profiling of Farmacista Honorati Persimmon Fruit (Diospyros kaki Thunb.) at Commercial Harvest and after Treatments for Astringency Removal
Source: Plants (Basel). 2024 Jun 26;13(13):1768. doi: 10.3390/plants13131768 (PMC11244366; doi:10.3390/plants13131768)
Supplement: Supplementary file 1 [file plants-13-01768-s001.zip › plants-3022441-supplementary.pdf]

Supplementary materials of "First data on the (poly)phenolic profiling of Farmacista Honorati persimmon fruit (*Diospyros kaki* Thunb.) at commercial harvest and after treatments for astringency removal" – Renai L. et al.

Table S1 – Moisture content of Farmacista Honorati (FH) samples, freeze-dried for 72 h. U, fruits collected at commercial harvest and untreated. CD, fruits treated with carbon dioxide. E, fruits treated with ethylene.

Sample	Moisture content (%)
FH-U	82
FH-CD	83
FH-E	80

MS parameters

The optimal source dependent parameters were as follows: Curtain Gas 40, CAD Gas Medium, Temperature 550 °C, Gas 1 60, Gas 2 40, Interface Heater ON and IonSpray Voltage −4500 V.

Table S2 – Optimized compound dependent parameters for the investigated compounds. Letter A refers to the quantifier transition, whereas letter B refers to qualifier transition. DP, declustering potential (V); EP, entrance potential (V); CE, collision energy (V); CXP, collision exit potential (V).

Compound	Precursor Ion (m/z)	Product Ion (m/z)	DP	EP	CE	CXP
GAL-A	169	125	-50	-9	-20	-11
GAL-B	169	137	-50	-9	-28	-15
PRO-A	153	109	-50	-9	-20	-10
PRO-B	153	108	-50	-9	-34	-10
NCHL-A	353	191	-70	-8	-25	-25
NCHL-B	353	179	-70	-8	-25	-20
p-HYD-A	137	93	-50	-8	-17	-8
p-HYD-B	137	60	-50	-8	-44	-10
PB1-A	577	289	-140	-7	-35	-30
PB1-B	577	407	-140	-7	-35	-20
EGC-A	305	125	-15	-8	-30	-13
EGC-B	305	137	-15	-8	-35	-17
CAT-A	289	245	-15	-9	-20	-20
CAT-B	289	109	-15	-9	-35	-10
CHL-A	353	191	-170	-13	-25	-20
CHL-B	353	85	-170	-13	-60	-10
ESC-A	177	133	-80	-7	-25	-15
ESC-B	177	105	-80	-7	-28	-25

CAF-A	179	135	-65	-10	-25	-12
CAF-B	179	134	-65	-10	-35	-10
PB2-A	577	289	-80	-7	-35	-30
PB2-B	577	407	-80	-7	-35	-25
VAN-A	167	123	-50	-8	-17	-12
VAN-B	167	108	-50	-8	-30	-11
CRY-A	353	173	-60	-8	-22	-15
CRY-B	353	179	-60	-8	-23	-16
EGCG-A	457	169	-20	-7	-30	-15
EGCG-B	457	125	-20	-7	-55	-15
PC1-A	865	407	-160	-7	-55	-30
PC1-B	865	289	-160	-7	-52	-25
EPI-A	289	245	-15	-9	-20	-20
EPI-B	289	109	-15	-9	-35	-10
GCG-A	457	169	-15	-8	-25	-15
GCG-B	457	125	-15	-8	-55	-15
p-COU-A	163	119	-50	-8	-20	-11
p-COU-B	163	93	-50	-8	-45	-10
ECG-A	441	169	-40	-8	-30	-15
ECG-B	441	125	-40	-8	-55	-15
SCO-A	191	176	-60	-7	-21	-16
SCO-B	191	104	-60	-7	-35	-14
PA2-A	575	285	-130	-8	-40	-25
PA2-B	575	289	-130	-8	-35	-20
CG-A	441	169	-15	-8	-30	-15
CG-B	441	125	-15	-8	-55	-15
FER-A	193	134	-50	-10	-20	-12
FER-B	193	149	-50	-10	-15	-14
SIN-A	223	149	-20	-8	-27	-15
SIN-B	223	164	-20	-8	-18	-20
SAL-A	137	93	-45	-7	-25	-10
SAL-B	137	65	-45	-7	-43	-20
QUE-GAL-A	463	300	-120	-8	-37	-15
QUE-GAL-B	463	271	-120	-8	-58	-15
EA-A	301	145	-180	-10	-50	-15
EA-B	301	229	-180	-10	-35	-20
DCQ-A	515	191	-40	-9	-40	-16
DCQ-B	515	353	-40	-9	-22	-15
QUE-RUT-A	609	300	-20	-5	-52	-15
QUE-RUT-B	609	271	-20	-5	-75	-15
QUE-GLU-A	463	300	-120	-8	-37	-15
QUE-GLU-B	463	271	-120	-8	-57	-15

PHL-GLU-A	435	273	-15	-7	-25	-20
PHL-GLU-B	435	167	-15	-7	-40	-15
QUE-RHA-A	447	300	-120	-8	-35	-15
QUE-RHA-B	447	271	-120	-8	-60	-15
KAM-RUT-A	593	285	-90	-6	-45	-25
KAM-RUT-B	593	255	-90	-6	-70	-20
KAM-GLU-A	447	284	-35	-8	-37	-12
KAM-GLU-B	447	255	-35	-8	-53	-15
QUE-A	301	151	-20	-7	-30	-14
QUE-B	301	179	-20	-7	-27	-17
LUT-A	285	133	-40	-10	-45	-15
LUT-B	285	151	-40	-10	-35	-15
PHL-A	273	167	-15	-9	-24	-15
PHL-B	273	123	-15	-9	-35	-10

Standard operative solutions and figures of merit of the LC-MS/MS method.

A multi-compound standard solution at a concentration of 10 mg/L of each compound was prepared by proper dilution of the standard stock solutions in MS grade methanol. For obtaining the calibration curves, a mixture of twenty-one standards in MS water was prepared and then injected (n=3) at seven different concentration levels; subsequently, the peak areas were plotted against the corresponding concentration values of each compound. The method for the analysis of individual persimmon polyphenols was evaluated in terms of i) instrumental limit of detection (ILOD) and quantitation (ILOQ), ii) method quantitation limit (MQL), ii) instrumental repeatability, linearity and range, iii) matrix effect (ME) and recovery (R%). **Table S3** shows the ILOD and ILOQ values, the repeatability data as RSD of peak area at 250 µg/L concentration value, and linearity data. The ILOD and the ILOQ values were determined by four successive injections of instrumental blanks and applying the following formula:

$$ILOD\ (ILOQ) = \frac{k \cdot \sigma_b}{m}$$

Where k is equal to $t_{1-\alpha}$=2.353 (n=3, α=0.05) for ILOD and equal to 10 for ILOQ, σ_b is standard deviation of the chromatographic area at the retention time of each analyte and m is the slope of the calibration curve. The ILOD values were included in the range 0.15-71.65 pg injected, corresponding to 0.03-14.33 µg/L; the ILOQ values were included in the range 0.50-304.55 pg injected corresponding to 0.10-60.91µg/L (5 µL injection volume). The MQLs values for all the target compounds ranged between 0.39 and 47.86 µg/100 g on a dry weight basis (d.w.). All the RSD values for the peak areas resulted lower than 5% or equal. Linearity was evaluated for all the compounds from the respective instrumental limit of quantitation (ILOQ) to 1000 µg/L, except for GAL and EA,

which were investigated from the ILOQ to 2000 µg/L. The linearity data were obtained by plotting the peak area of each analyte vs. the corresponding concentration and the coefficients of determination ranged from R^2= 0.9901 to R^2= 0.9994.

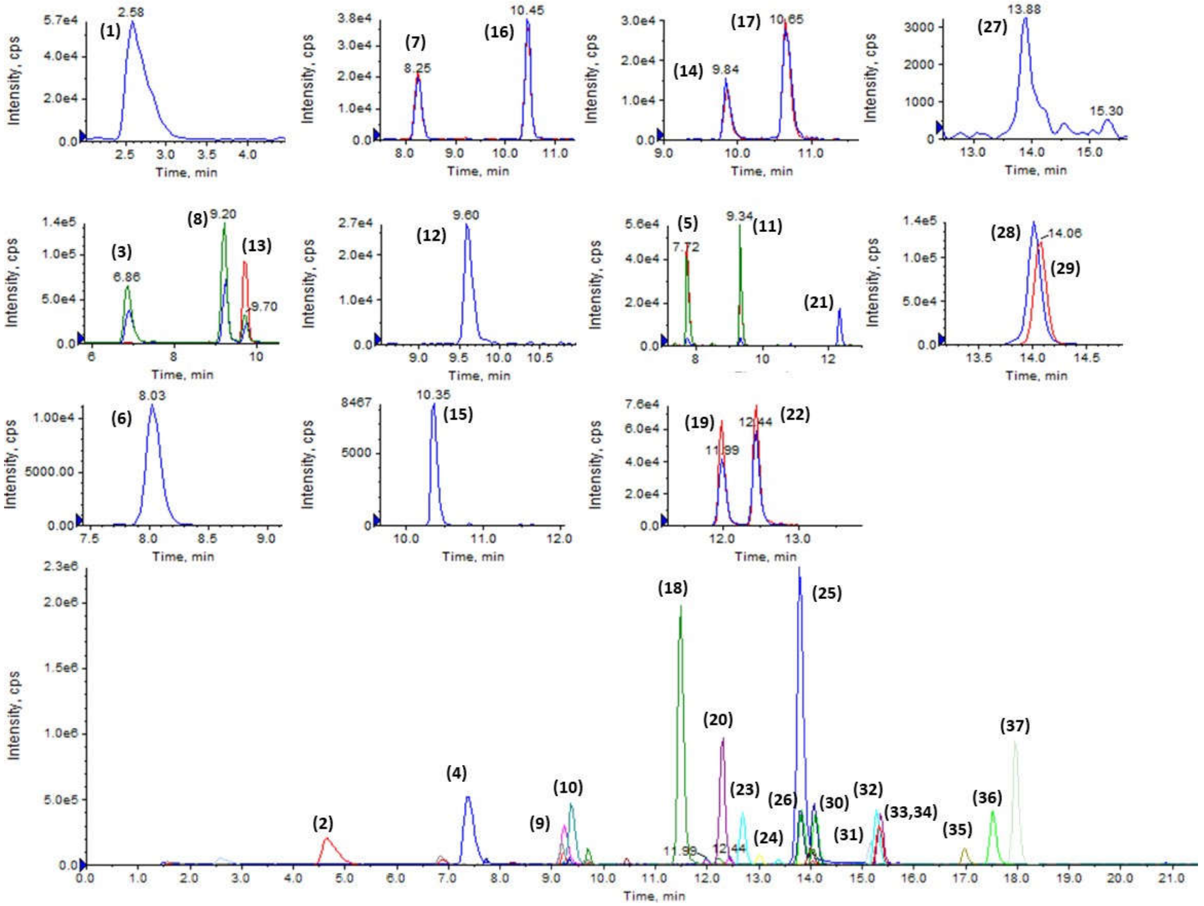

Figure S1 – Typical MRM reconstructed LC–MS/MS chromatogram of a standard mixture (100 µg/L) of the investigated (poly)phenols. (1) GAL (2) PRO (3) NCHL (4) p-HYD (5) PB1 (6) EGC (7) CAT (8) CHL (9) ESC (10) CAF (11) PB2 (12) VAN (13) CRY (14) EGCG (15) PC1 (16) EPI (17) GCG (18) p–COU (19) ECG (20) SCO (21) PA2 (22) CG (23) FER (24) SIN (25) SAL (26) QUE-GAL (27) EA (28) DCQ (29) QUE-RUT (30) QUE-GLU (31) PHL-GLU (32) QUE-RHA (33) KAM-RUT (34) KAM-GLU (35) QUE (36) LUT (37) PHL. The meaning of the abbreviations of the compounds is reported in Paragraph 3.1.

Table S3 – Values of instrumental ILOD and ILOQ (μg/L), %RSD values of peak area (250 μg/L), linearity data: linearity range (μg/L) and coefficient of determination (R^2).

Compound	ILOD	ILOQ	% RSD	Range	R^2
GAL	3.1	13.4	3.07	13.4-2000	0.9975
PRO	0.25	1.10	2.92	1.1-1000	0.9993
NCHL	2.20	9.35	4.85	9.35-1000	0.9984
p-HYD	0.49	2.09	1.60	2.09-1000	0.9974
PB1	0.56	2.40	4.35	2.4-1000	0.9983
EGC	1.15	4.88	4.17	4.88-1000	0.9956
CAT	2.34	9.93	0.73	9.93-1000	0.9994
CHL	3.6	15.5	2.19	15.5-1000	0.9980
ESC	1.61	6.84	1.82	6.84-1000	0.9955
CAF	1.0	4.20	3.73	4.2-1000	0.9968
PB2	0.14	0.60	4.47	0.6-1000	0.9979
VAN	3.73	15.80	4.74	15.80-1000	0.9912
CRY	0.80	3.38	4.78	3.38-1000	0.9984
EGCG	0.65	2.75	2.76	2.75-1000	0.9963
PC1	2.71	12.0	2.51	12.0-1000	0.9929
EPI	3.37	14.33	2.01	14.33-1000	0.9991
GCG	0.69	2.97	3.19	2.97-1000	0.9926
p-COU	0.73	3.10	3.04	3.1-1000	0.9960
ECG	0.17	0.74	3.30	0.74-1000	0.9989
SCO	0.27	1.15	4.93	1.15-1000	0.9910
PA2	1.10	4.68	4.16	4.68-1000	0.9975
CG	0.30	1.32	4.85	1.32-1000	0.9976
FER	0.03	0.10	5.29	0.10-1000	0.9976
SIN	1.28	5.45	2.83	5.45-1000	0.9974
SAL	0.23	0.98	0.67	0.98-1000	0.9982
QUE-GAL	0.28	1.18	4.77	1.18-1000	0.9957
EA	9.5	51.56	4.53	60.91-2000	0.9901
DCQ	0.16	0.69	4.30	0.69-1000	0.9990
QUE-RUT	0.39	1.66	4.96	1.66-1000	0.9960
QUE-GLU	0.20	0.87	4.49	0.87-1000	0.9961
PHL-GLU	0.25	1.07	2.19	1.07-1000	0.9976
QUE-RHA	1.15	4.89	2.48	4.89-1000	0.9929
KAM-RUT	0.24	1.04	3.19	0.51-1000	0.9986
KAM-GLU	0.07	0.30	3.06	0.30-1000	0.9970
QUE	0.11	0.47	3.37	0.47-1000	0.9973
LUT	0.45	1.49	1.19	1.49-1000	0.9977
PHL	0.65	2.75	3.92	2.75-1000	0.9945
